# Heterotrophic Thaumarchaea with Small Genomes Are Widespread in the Dark Ocean

**DOI:** 10.1128/mSystems.00415-20

**Published:** 2020-06-16

**Authors:** Frank O. Aylward, Alyson E. Santoro

**Affiliations:** a Department of Biological Sciences, Virginia Tech, Blacksburg, Virginia, USA; b Department of Ecology, Evolution and Marine Biology, University of California, Santa Barbara, California, USA; Lawrence Berkeley National Laboratory

**Keywords:** *Thaumarchaeota*, marine archaea, TACK, PQQ-dehydrogenase, RuBisCO

## Abstract

The *Thaumarchaeota* is a diverse archaeal phylum comprising numerous lineages that play key roles in global biogeochemical cycling, particularly in the ocean. To date, all genomically characterized marine thaumarchaea are reported to be chemolithoautotrophic ammonia oxidizers. In this study, we report a group of putatively heterotrophic marine thaumarchaea (HMT) with small genome sizes that is globally abundant in the mesopelagic, apparently lacking the ability to oxidize ammonia. We assembled five HMT genomes from metagenomic data and show that they form a deeply branching sister lineage to the ammonia-oxidizing archaea (AOA). We identify this group in metagenomes from mesopelagic waters in all major ocean basins, with abundances reaching up to 6% of that of AOA. Surprisingly, we predict the HMT have small genomes of ∼1 Mbp, and our ancestral state reconstruction indicates this lineage has undergone substantial genome reduction compared to other related archaea. The genomic repertoire of HMT indicates a versatile metabolism for aerobic chemoorganoheterotrophy that includes a divergent form III-a RuBisCO, a 2M respiratory complex I that has been hypothesized to increase energetic efficiency, and a three-subunit heme-copper oxidase complex IV that is absent from AOA. We also identify 21 pyrroloquinoline quinone (PQQ)-dependent dehydrogenases that are predicted to supply reducing equivalents to the electron transport chain and are among the most highly expressed HMT genes, suggesting these enzymes play an important role in the physiology of this group. Our results suggest that heterotrophic members of the *Thaumarchaeota* are widespread in the ocean and potentially play key roles in global chemical transformations.

**IMPORTANCE** It has been known for many years that marine *Thaumarchaeota* are abundant constituents of dark ocean microbial communities, where their ability to couple ammonia oxidation and carbon fixation plays a critical role in nutrient dynamics. In this study, we describe an abundant group of putatively heterotrophic marine *Thaumarchaeota* (HMT) in the ocean with physiology distinct from those of their ammonia-oxidizing relatives. HMT lack the ability to oxidize ammonia and fix carbon via the 3-hydroxypropionate/4-hydroxybutyrate pathway but instead encode a form III-a RuBisCO and diverse PQQ-dependent dehydrogenases that are likely used to conserve energy in the dark ocean. Our work expands the scope of known diversity of *Thaumarchaeota* in the ocean and provides important insight into a widespread marine lineage.

## Abstract (precis)

It has been known for many years that marine *Thaumarchaeota* are abundant constituents of dark ocean microbial communities, where their ability to couple ammonia oxidation and carbon fixation plays a critical role in nutrient dynamics. In this study, we describe an abundant group of putatively heterotrophic marine *Thaumarchaeota* (HMT) in the ocean with physiology distinct from those of their ammonia-oxidizing relatives. HMT lack the ability to oxidize ammonia and fix carbon via the 3-hydroxypropionate/4-hydroxybutyrate pathway but instead encode a form III-a RuBisCO and diverse PQQ-dependent dehydrogenases that are likely used to conserve energy in the dark ocean. Our work expands the scope of known diversity of *Thaumarchaeota* in the ocean and provides important insight into a widespread marine lineage.

## INTRODUCTION

Archaea represent a major fraction of the microbial biomass on Earth and play key roles in global biogeochemical cycles (1, 2). Historically the *Crenarchaeota* and *Euryarchaeota* were among the most well-studied archaeal phyla owing to the preponderance of cultivated representatives in these groups, but recent advances have led to the discovery of numerous additional phyla in this domain (3, 4). Among the first of these newly described phyla was the *Thaumarchaeota* (5), forming part of a superphylum that, together with the *Crenarchaeota* and candidate phyla “*Candidatus* Aigarchaeota” and “*Candidatus* Korarchaeota,” is known as TACK (6). Coupled with recent discoveries of other lineages, such as the “*Candidatus* Bathyarchaeota” and Asgard superphylum (7, 8), the archaeal tree has grown substantially. Moreover, intriguing findings within the DPANN group, an assortment of putatively early branching lineages, have shown that archaea with small genomes and highly reduced metabolism are common in diverse environments (9, 10). Placement of both the DPANN and the TACK superphyla remains controversial (11, 12), highlighting the need for additional genomes from diverse archaea.

Members of the *Thaumarchaeota* are particularly important contributors to global biogeochemical cycles, in part because this group comprises all known ammonia-oxidizing archaea (AOA) (13), chemolithoautotrophs carrying out the first step of nitrification, a central process in the nitrogen cycle. Deep waters beyond the reach of sunlight comprise the vast majority of the volume of the ocean (14); in these habitats, thaumarchaea can comprise up to 30% of all cells and are critical drivers of primary production and nitrogen cycling (15, 16). Although much research on this phylum has focused on AOA, recent work has begun to show that basal-branching groups or close relatives of the *Thaumarchaeota* are broadly distributed in the biosphere and have metabolisms distinct from those of their ammonia-oxidizing relatives. This includes the “*Candidatus* Aigarchaeota” as well as several early-branching *Thaumarchaeota*, which have been discovered in hot springs, anoxic peats, and deep subsurface environments (17–20).

In this study, we characterize a group of heterotrophic marine thaumarchaea (HMT) with a small genome size that is broadly distributed in deep ocean waters across the globe. We show that although this group is a sister lineage to the AOA, it does not contain the molecular machinery for ammonia oxidation or the 3-hydroxypropionate/4-hydroxybutyrate (3HP/4HB) cycle for carbon fixation but instead encodes multiple pathways comprising a putatively chemoorganoheterotrophic lifestyle, including numerous pyrroloquinoline quinone (PQQ)-dependent dehydrogenases and a divergent form III-a RuBisCO. Our work describes non-AOA members of the *Thaumarchaeota* that are ubiquitous in the dark ocean and potentially important contributors to carbon transformations in this globally important habitat.

## RESULTS AND DISCUSSION

### Phylogenomics and biogeography of the HMT.

We generated metagenome assembled genomes (MAGs) from 4 hadopelagic metagenomes from the Pacific (bioGEOTRACES samples) and 9 metagenomes from 750- to 5,000-m depth in the Atlantic (DeepDOM cruise). After screening and scaffolding the resulting MAGs, we retrieved 5 high-quality HMT MAGs with completeness of >75% and contamination of <2% (Table 1; see Materials and Methods for details). All MAGs shared high average nucleotide identity (ANI), reflecting low genomic diversity within this group irrespective of their ocean basin of origin (99.6% ANI between the Pacific and Atlantic MAGs, minimum of 97% ANI overall). Using the Genome Taxonomy Toolkit (21, 22), we classified the MAGs into the order *Nitrososphaerales* within the class *Nitrososphaeria*, indicating their evolutionary relatedness to ammonia-oxidizing archaea (AOA). We also performed a phylogenetic analysis of the HMT MAGs together with reference genomes from the *Thaumarchaeota* and “*Candidatus* Aigarchaeota” using a whole-genome phylogeny approach, which suggests the placement of the HMT as a sister clade to the AOA (see Fig. S1 in the supplemental material). The reference genome UBA57, which was previously assembled from a Mid-Cayman Rise metagenome as part of a large-scale genomes-from-metagenomes workflow (23), also fell within the HMT group, as did the ASW8 MAG, which was recently assembled from a Monterey Bay metagenome (24) (Table 1 and Fig. S1).

**TABLE 1:** HMT *Thaumarchaeota* MAG statistics

MAG ID	Bin size (kbp)	Source or reference	Completeness (%)		Contamination (CheckM)
			CheckM	Rinke^*a*^	
HMT_ATL	837.8	This study	98.1	89.4	1.5
HMT_PAC	812.1	This study	96.1	88.5	0.97
HMT_AAIW	697.5	This study	78.1	72.6	0.97
HMT_NADW	670.8	This study	76.6	75.2	0
HMT_AABW	814.8	This study	88.8	77.0	0.97
ASW8	996.5	24	97.1	92.0	2.91
UBA57	613.3	23	62.7	66.4	0

a Estimates made using the Rinke et al. (38) marker set (see Materials and Methods for details).

10.1128/mSystems.00415-20.1 FIG S1 Concatenated maximum-likelihood phylogeny based on 30 highly conserved marker genes including *Thaumarchaeota* genomes with completeness of >50% (referred to as the full genome set; see Materials and Methods for details). The HMT clade is shown in blue. Black circles denote nodes with >80% bootstrap support, inferred using 1,000 ultrafast bootstraps in IQ-TREE. The collapsed node contains 147 genomes of ammonia-oxidizing archaea (AOA). Download FIG S1, PDF file, 0.03 MB. Copyright © 2020 Aylward and Santoro. 2020 Aylward and Santoro This content is distributed under the terms of the Creative Commons Attribution 4.0 International license.

All HMT MAGs we generated carried a full-length 16S rRNA gene; therefore, we constructed a phylogenetic tree based on this marker to examine if previous surveys had identified the HMT lineage (Fig. S2). We found that the 16S rRNA gene sequences of the HMT MAGs are part of a broader clade that has been observed previously in diverse oceanic provinces, including the Puerto Rico Trench (25), Arctic Ocean (26), Monterey Bay (27, 28), the Suiyo Seamount hydrothermal vent water (29, 30), the Juan de Fuca Ridge (31), and deep waters of the North Pacific Subtropical Gyre (28, 32), Ionian Sea (33), and North Atlantic (34). Previous work has referred to this lineage as the pSL12-related group (28, 35) and noted that it forms a sister clade to the ammonia-oxidizing *Thaumarchaeota*, consistent with our 16S rRNA gene and concatenated marker protein phylogenies (Fig. S1 and S2). Three fosmids were previously sequenced from this lineage (30) (AD1000-325-A12, KM3-153-F8, and AD1000-23-H12 in Fig. S2), and their corresponding 16S rRNA gene sequences have 85 to 95% identity to the 16S rRNA genes of our HMT MAGs. Moreover, we compared the amino acid sequences in the fosmids and found they have 41 to 72% average amino acid identity (AAI) to those of our HMT MAGs, suggesting that considerable genomic variability exists within this group in different marine habitats. Overall, the occurrence of sequences from this clade in such diverse marine environments suggests that the HMT lineage represents a broadly distributed group that, although observed in numerous previous studies, has remained poorly characterized.

10.1128/mSystems.00415-20.2 FIG S2 Maximum-likelihood tree of the HMT using 16S rRNA genes and references available in NCBI and the Ribosomal Database Project. Black circles denote nodes with >80% bootstrap support, inferred using 1,000 ultrafast bootstraps in IQ-TREE. 16S rRNA gene sequences from the 5 MAGs generated in this study are colored blue. Download FIG S2, PDF file, 0.03 MB. Copyright © 2020 Aylward and Santoro. 2020 Aylward and Santoro This content is distributed under the terms of the Creative Commons Attribution 4.0 International license.

**FIG 1:**
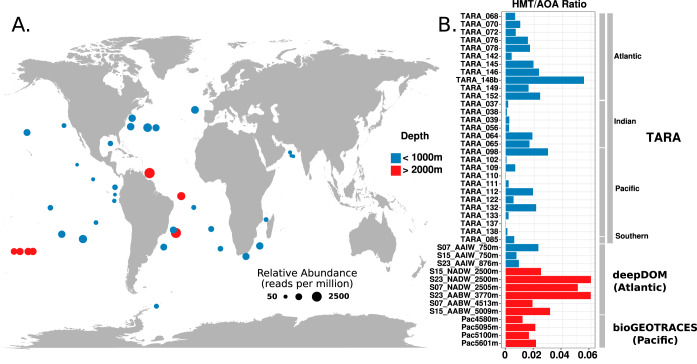
**(**A) Relative abundance of the HMT lineage in metagenomic data collected from across the global ocean at depths ranging from 250 to 1,000 m (blue) and >2,000 m (red). HMT relative abundances were calculated by mapping metagenomic reads against a nonredundant set of HMT proteins and are presented in units of reads per million. (B) Relative abundances of HMT versus AOA in different metagenomic samples. Values are the ratio of HMT to AOA, calculated by mapping reads to single-copy marker genes (see Materials and Methods for details). Bars in red denote metagenomic samples taken at depths of >2,000 m, while blue bars denote depths of <1,000 m.

To investigate the biogeography of HMT, we leveraged our genomic data to assess the relative abundance of this group in mesopelagic Tara Oceans metagenomes from 29 locations (depths of 250 to 1,000 m) and 13 metagenomes from the bioGEOTRACES samples and DeepDOM cruises (depths of 750 to 5,601 m) (Fig. 1A). For this, we generated a nonredundant set of HMT proteins from the 5 MAGs we assembled together with the UBA57 and ASW8 genomes, and we mapped reads to these protein sequences with a translated search implemented in LAST (36) (amino acid identity, >90%; see Materials and Methods). Reads mapping to the HMT were detected in all of the metagenomes we analyzed, demonstrating their global presence in waters of the Atlantic, Pacific, and Indian Oceans at depths ranging from 250 to 5,601 m (Fig. 1A). Given the dominance of chemolithoautotrophic AOA in the dark ocean, we sought to compare the relative abundance of HMT to AOA in metagenomic samples. To do this, we assessed the number of metagenomic reads mapping to the HMT-specific RuBisCO large subunit protein using a translated LAST search and compared the results to the number of reads that mapped to a set of thaumarchaeal AmoA proteins (see Materials and Methods for details), with abundances normalized by gene length. This approach estimated that HMT can reach abundances of up to 6% of those of their AOA relatives (Fig. 1B) and had mean abundances of 1.4% of that of AOA (Data Set S1). These results suggest that HMT are globally distributed but comprise a relatively small fraction of the thaumarchaeal population in the dark ocean compared to AOA. A recent study identified the presence of the ASW8 MAG genome in Monterey Bay (depths of 5 to 500 m) and reported that it represented abundances of <0.5% of the total thaumarchaeal population (24), further suggesting that this group comprises a relatively small fraction of total archaea in shallow waters.

10.1128/mSystems.00415-20.6 DATA SET S1 Raw results from metagenome and metatranscriptome read-mapping analyses. Download Data Set S1, XLSX file, 0.09 MB. Copyright © 2020 Aylward and Santoro. 2020 Aylward and Santoro This content is distributed under the terms of the Creative Commons Attribution 4.0 International license.

**FIG 2:**
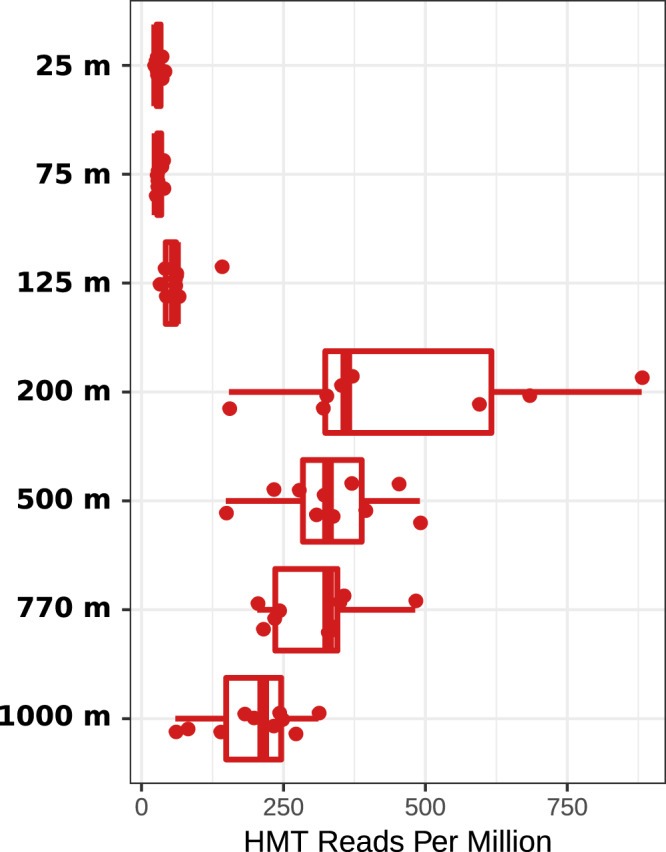
Abundance of HMT at Station ALOHA from 25 to 1,000 m. Metagenomic reads from samples collected from 2010 to 2011 were mapped against a nonredundant set of HMT proteins (see Materials and Methods for details). Relative abundance units are given in reads mapped per million.

To investigate the distribution of HMT across different depths in the water column, we assessed their relative abundance in metagenomes from Station ALOHA in the North Pacific Subtropical Gyre. We analyzed metagenomes sampled from 10 cruises of the Hawaii Ocean time series in 2010 and 2011 that correspond to depths of 25 m, 75 m, 125 m, 500 m, 770 m, and 1,000 m and were described previously (37). These results show that HMT are not detectable in surface waters (25 to 75 m) but can be detected deeper in the water column, albeit with very low abundance at 125 m (Fig. 2 and Fig. S3; full mapping results are in Data Set S1). Interestingly, HMT relative abundance appears to peak at 200 m; this is consistent with a previous study that assessed pSL12 abundance using quantitative PCR across a large region of the central Pacific Ocean, which found pSL12-like organisms present below the euphotic zone and tended to have the highest abundance near 200 m (35). Comparison of the relative abundance of HMT and AOA indicates that HMT comprise, on average, 1% of the thaumarchaeal community at Station ALOHA at depths of 200 to 1,000 m, consistent with similar values we obtained from the Tara, bioGEOTRACES, and DeepDOM samples (Data Set S1). Overall, the presence of HMT in metagenomes and 16S rRNA gene libraries sampled across a wide range of depths and geographic locations indicates this group is ubiquitous in the dark ocean.

10.1128/mSystems.00415-20.3 FIG S3 Depth profile comparisons of HMT (red) and AOA (blue) at Station ALOHA based on marker gene mapping (top) and whole-genome mapping (bottom). The line in the boxplot denotes the median value. Details regarding the mapping can be found in Materials and Methods. Raw data can be found in Data Set S1. Download FIG S3, PDF file, 0.07 MB. Copyright © 2020 Aylward and Santoro. 2020 Aylward and Santoro This content is distributed under the terms of the Creative Commons Attribution 4.0 International license.

### Genome reduction in the HMT lineage.

The HMT MAGs we assembled are all between 671 and 838 kbp in size, while the ASW8 MAG is 997 kbp and the UBA57 MAG is 613.3 kbp (Table 1 and Data Set S2). Because the MAGs are predicted to be mostly complete and contain little contamination, we estimated the size of complete HMT genomes. By extrapolating complete genome sizes using the CheckM completeness and contamination estimates, the predicted complete genome sizes of this group range between 837 and 997 kbp (see Materials and Methods for details; full genome statistics available in Data Set S2), which would be notably small genomes for planktonic marine archaea. To provide an additional evaluation of the completeness of the HMT genomes, we performed further analysis by leveraging an alternative set of 113 highly conserved archaeal marker genes, which are part of a larger set previously used by Rinke et al. to assess completeness in archaeal genomes (38) (see Materials and Methods for details on this analysis). Using the Rinke marker set, we generally recovered lower completeness estimates (66 to 92% as opposed to the 62 to 98% estimated by CheckM; Table 1), which suggests that complete genome sizes fall within the 891- to 1,050-kbp range. Given the ASW8 MAG is already 997 kbp in length, it is likely HMT genomes fall on the higher end of this range, although further work assessing complete genomes will be necessary to determine this definitively. Regardless, these findings indicate that the HMT contain reduced genomes that are smaller than those of any previously reported *Thaumarchaeota* but slightly larger than those of some DPANN archaea (9, 39).

10.1128/mSystems.00415-20.7 DATA SET S2 Statistics for the HMT and reference genomes used in this study. Download Data Set S2, XLSX file, 0.1 MB. Copyright © 2020 Aylward and Santoro. 2020 Aylward and Santoro This content is distributed under the terms of the Creative Commons Attribution 4.0 International license.

**FIG 3:**
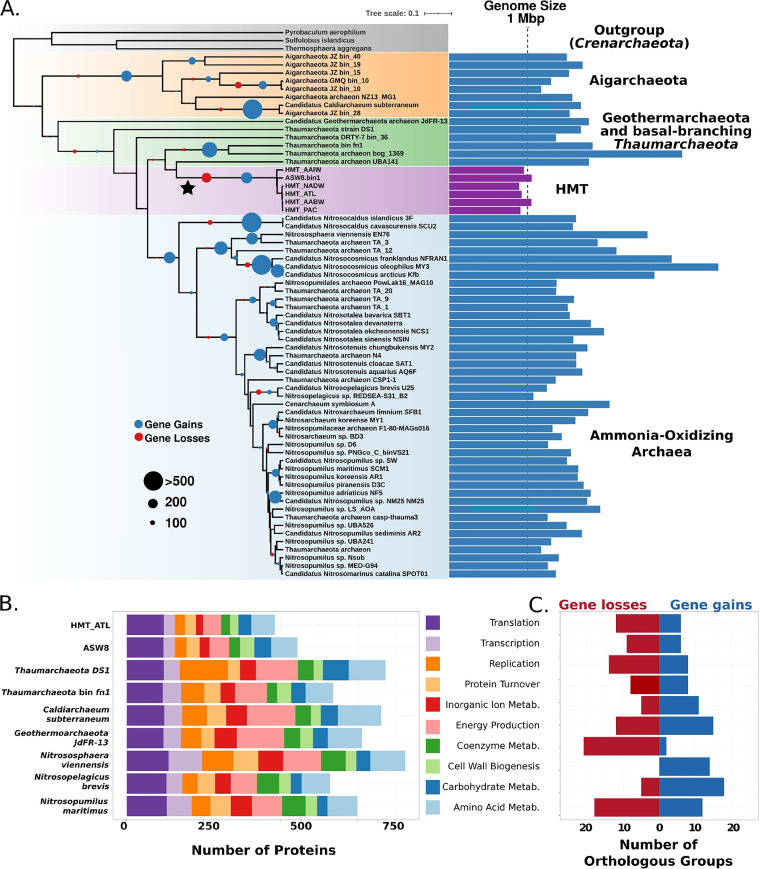
Phylogeny and gene loss analysis. (A) Maximum-likelihood phylogeny of high-quality reference genomes based on a concatenation of 30 marker genes. The phylogeny was generated in IQ-TREE with the C60 substitution model (see Materials and Methods for details). HMT genome sizes are colored purple. Complete genome sizes were estimated for incomplete genomes using completeness and contamination estimates (see Materials and Methods). Circles at the nodes provide the number of estimated gains and losses of orthologous groups. (B) COG composition of two HMT genomes and select high-quality reference genomes. Only genes annotated to select categories are provided; full annotations for all genomes are available in Data Set S3. (C) Functional analysis of the OGs gained and lost on the branch leading to the HMT (star in panel A).

We calculated orthologous groups (OGs) between the HMT MAGs and a set of high-quality reference archaeal genomes (estimated completeness of >90% and contamination of <2% by CheckM; referred to as the high-quality reference genome set; Data Set S2), resulting in a total of 28,166 OGs (Data Set S3). We then performed ancestral state reconstructions on the OGs to distinguish between those that were lost by the HMT and those that were gained by other lineages. This analysis estimated that 180 OGs were lost on the branch leading to the HMT (Fig. 3A), which is the largest single incidence of gene loss in our analysis. Compared to other reference *Thaumarchaeota* genomes, the HMT carried markedly fewer genes involved in several broad functional categories, including energy metabolism, inorganic ion metabolism, coenzyme metabolism, and amino acid metabolism (Fig. 3B). Moreover, many genes in these categories appear to have been lost specifically in the HMT lineage, indicating this group has undergone marked genome reduction, similar to many other marine lineages of bacteria and archaea (40). The HMT genomes lacked several highly conserved metabolic genes, including succinate dehydrogenase subunits, genes in tetrapyrrole biosynthesis (*hemABCD*) and riboflavin metabolism (*ribBD*, *ribE*, and *ribH*), and genes for cobalamin biosynthesis. The absence of cobalamin biosynthesis is coincident with the acquisition of the vitamin B_12_-independent methionine synthesis pathway (*metE*) and is in sharp contrast to AOA, which have been postulated to be major producers of this coenzyme (41, 42). Although these findings indicate that the HMT genomes have gone through a period of genome reduction, several genes have also been acquired in this lineage, most notably those involved in carbohydrate metabolism and cell wall biogenesis (Fig. 3C), including several glycosyl transferases, pyrroloquinoline-quinone (PQQ)-dependent dehydrogenases, a phosphoglucosamine mutase, and several sugar dehydratases.

10.1128/mSystems.00415-20.8 DATA SET S3 Annotations for the orthologous groups, HMT nonredundant protein set, and individual MAGs. Results are for the homology searches of HMT PQQ-dependent dehydrogenases when queried against NCBI databases. Download Data Set S3, XLSX file, 3.6 MB. Copyright © 2020 Aylward and Santoro. 2020 Aylward and Santoro This content is distributed under the terms of the Creative Commons Attribution 4.0 International license.

Unlike the small genomes of the bacterial candidate phyla radiation (CPR) and DPANN lineages (9), HMT genomes lack evidence to suggest they are symbionts of other cells. Like the AOA, the HMT genomes contain genes for both the FtsZ-based cell division system and a Cdv-based cell division cycle (CdvA, CdvB, and CdvC), although only the latter has been functionally confirmed in AOA (43). Genes for the synthesis of all amino acids are present in the same complement as other marine *Thaumarchaeota* (44), with the exception of an alternate lysine biosynthesis pathway found primarily in methanogens (45). Although knowledge of the biosynthetic pathway for crenarchaeol and other glycerol dibiphytanyl glycerol tetraether (GDGT) membrane lipids is incomplete, HMT appear to have the same identifiable components present in other *Thaumarchaeota* (46, 47). Together, this genomic evidence suggests that the HMT have retained a free-living lifestyle despite their genome reduction.

### Predicted metabolism of the HMT.

**FIG 4:**
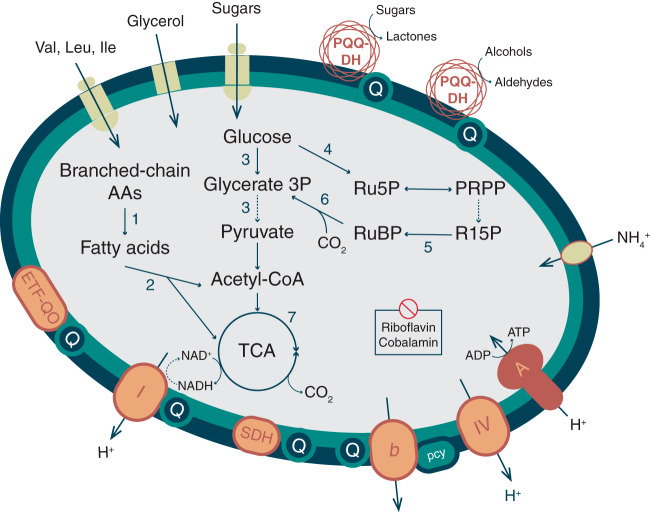
Hypothesized central carbon metabolism, electron transport chain, and selected transport capabilities in HMT. Dashed arrows indicate pathways with uncertain enzymology. Pathways: 1, branched-chain alpha-ketoacid dehydrogenase complex; 2, beta oxidation; 3, glycolysis; 4, nonoxidative pentose phosphate pathway; 5, ribose 1,5 bisphosphate isomerase; 6, ribulose bisphosphate carboxylase (RuBisCO); 7, tricarboxylic acid cycle. Annotations for all genes hypothesized to encode proteins in the numbered pathways and all depicted respiratory complexes are included in Data Set S4. Abbreviations: AAs, amino acids; ETF-QO, electron transfer flavoprotein-quinol oxidase; Ile, isoleucine; pcy, plastocyanin; PQQ-DH, pyrroloquinoline-quinone-dependent dehydrogenase; PRPP, phosphoribosyl pyrophosphate; Leu, leucine; Q, quinone pool; R15P, ribose 1,5-bisphosphate; Ru5P, ribose 5-phosphate; RuBP, ribulose 1,5-bisphosphate; SDH, succinate:fumarate dehydrogenase; TCA, tricarboxylic acid cycle; Val, valine. Figure design is modeled after figures from reference 9.

HMT are putative aerobic chemoorganoheterotrophs, as evidenced by the presence of a full glycolysis pathway, the beta-oxidation pathway for fatty acid degradation, and a nearly complete tricarboxylic acid (TCA) cycle that can deliver reducing equivalents to a complete aerobic respiratory chain (Fig. 4; curated annotations for all genes discussed in this section can be found in Data Set S4). No glycoside hydrolases were detected, suggesting they do not metabolize complex polysaccharides, but they do encode an ABC sugar transporter that may take up simple oligo- and monosaccharides as well as a glycerol transporter. As with AOA genomes, the final pyruvate generating step of glycolysis is uncertain (PEP to pyruvate); however, the HMT genomes contain a putative phosphenolpyruvate synthetase, shown to be bidirectional in some archaea (48).

10.1128/mSystems.00415-20.9 DATA SET S4 Curated metabolic reconstruction of HMT pathways. Download Data Set S4, XLSX file, 0.06 MB. Copyright © 2020 Aylward and Santoro. 2020 Aylward and Santoro This content is distributed under the terms of the Creative Commons Attribution 4.0 International license.

Four genes originally annotated as a pyruvate dehydrogenase complex also share homology with the branched-chain α-ketoacid dehydrogenase complex (BCKDC) and were annotated as such by the MaGe pipeline (see Materials and Methods). This complex could allow energy conservation from branched-chain amino acids by converting them to fatty acids, which subsequently would be oxidized by the beta-oxidation pathway, generating reducing equivalents, acetyl-coenzyme A (CoA) and/or propionyl-CoA. Genes encoding both the membrane and binding components of an ABC transporter for branched amino acid transport are present in the MAGs, further facilitating these as energy sources. In addition to the transport of amino acids, the HMT genomes encode several zinc- and cobalt-containing metallopeptidases, including a leucyl aminopeptidase that could additionally furnish amino acids to this pathway. This metabolic module also apparently includes an electron transfer flavoprotein (ETF), known to serve as an electron carrier in beta-oxidation, and a membrane-bound ETF-quinol oxidase that could deliver electrons to the quinone pool (49).

The HMT respiratory chain differs in several interesting ways from that previously identified in the *Thaumarchaeota*. The MAGs encode a full complex I (NUO), but, like the AOA (50), lack subunits NuoEFG, thought to facilitate the transfer of electrons from NADH, making the electron donor to this complex uncertain. HMT complex I belongs to clade 2 of the recently described 2M form of NUO, containing a duplication of the NuoM subunit, hypothesized to facilitate the translocation of an additional proton per electron (51). We identified the flavoprotein subunit of a putative succinate dehydrogenase (SDH; complex II) but were unable to identify the Fe-S or transmembrane components. While AOA contain a four-subunit version of SDH with these genes colocated in the genome, HMT potentially utilize an alternative form similar to that found in the nitrite-oxidizing bacteria *Nitrospira* (52). A putative cytochrome *b*-like complex III with a homolog in the *Nitrosphaera gargensis* genome was identified (BLASTP, 53% identity) that could accept electrons from the quinone pool. Like the AOA (53), HMT appear to utilize blue copper domain-containing plastocyanin-like proteins for electron transport, with five such genes in the HMT_ATL MAG. One of these proteins could specifically serve a role as an electron carrier between complex III and IV, as has been hypothesized in the AOA (46, 54). The heme-copper oxidase (HCO) putatively serving as a terminal oxidase in complex IV is divergent from that present in AOA and contains a distinct subunit III, identified as one of the genes gained by HMT in our ancestral state reconstructions. Several classification schemes have been proposed for the HCO superfamily (55, 56); the HMT version appears most similar to the A2 type, characterized by a tyrosine residue in one of the two proton-transporting channels (57), and is rare in archaeal genomes. There is no evidence that the HMT can use other terminal electron acceptors, lacking identifiable nitrate, nitrite, or sulfate reductases.

The HMT genomes encode a single A-type ATPase similar to that encoded in neutrophilic AOA. A recent study showed that horizontal transfer has shaped the distribution of H^+^-pumping ATPase operons in *Thaumarchaeota*, with some deepwater or acidophilic lineages convergently acquiring a distinct V-type-like ATPase that potentially provides a fitness benefit in extreme environments (58). We performed a phylogenetic analysis of subunits A and B of this complex that demonstrated it has a phylogenetic signal consistent with the concatenated marker gene tree for this group, with the HMT forming a sister clade to neutrophilic AOA (Fig. S4). This indicates that despite the unusual genomic features of this group, the ATPase of the HMT has not been shaped by horizontal transfer in the same way as some hadopelagic or acidophilic thaumarchaea and likely functions analogously to that of neutrophilic AOA.

10.1128/mSystems.00415-20.4 FIG S4 Maximum-likelihood phylogeny of the ATPase subunits present in the HMT genomes and available *Thaumarchaeota* references. The phylogeny is based on a concatenated alignment of ATPase subunits A and B. Black circles denote nodes with >80% bootstrap support, inferred using 1,000 ultrafast bootstraps in IQ-TREE (see Materials and Methods for details). Download FIG S4, PDF file, 0.04 MB. Copyright © 2020 Aylward and Santoro. 2020 Aylward and Santoro This content is distributed under the terms of the Creative Commons Attribution 4.0 International license.

Given the small genomes of the HMT lineage, it is notable that they encoded multiple PQQ-dependent dehydrogenases (quinoproteins), which are rarely found in archaeal genomes. Individual MAGs encoded between 4 and 19 individual PQQ-dependent dehydrogenases, and we generated a nonredundant set of proteins from 7 HMT MAGs (the 5 we present here in addition to ASW8 and UBA57) that contained 21 total distinct PQQ-dehydrogenase variants (Data Set S3). PQQ-dependent dehydrogenases potentially target diverse carbon compounds and deliver reducing equivalents directly to the electron transport chain (ETC) through ubiquinone or cytochrome *c* (59–61) without the need for energetic transport across the inner membrane (62). We hypothesize that PQQ-dependent dehydrogenases serve a similar function in HMT, but, lacking cytochrome *c*, funnel reducing equivalents to the quinone pool or potentially one of the blue copper proteins (Fig. 4). An analogous use of alcohol dehydrogenases to support ATP synthesis, but not biomass production, from diverse alcohols was shown for members of the SAR11 clade of alphaproteobacteria (63), also abundant in low-nutrient environments. The HMT genomes also encode a PQQ synthase and can likely produce the cofactor for these enzymes (Data Set S4).

**FIG 5:**
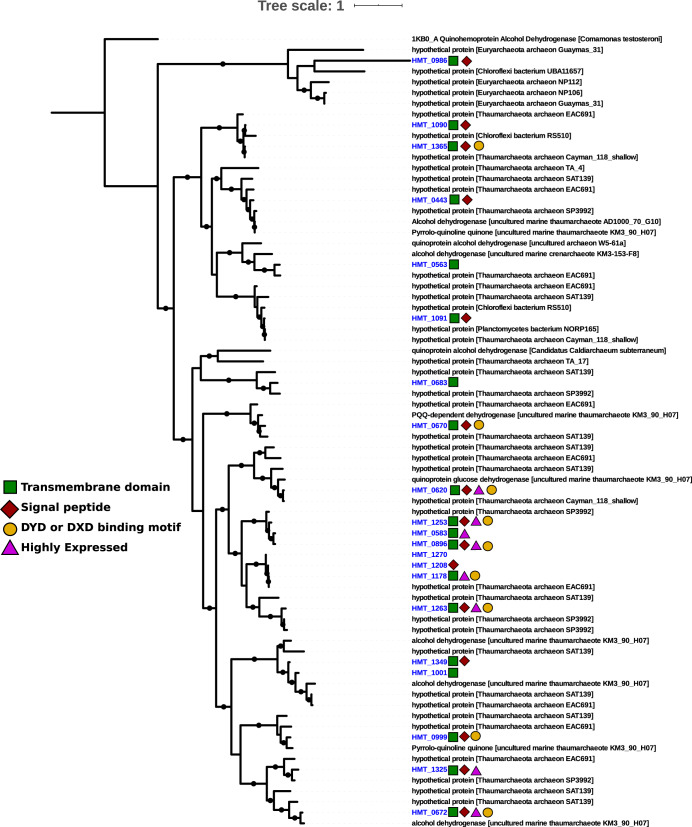
Maximum-likelihood phylogeny of 21 HMT PQQ-dependent dehydrogenase families identified in the nonredundant set of HMT proteins (blue) together with reference sequences in the NCBI NR database. Colors symbols indicate the presence of transmembrane domains, signal peptides, and binding motifs in the HMT enzymes. Enzymes that were among the top 40 most highly expressed genes in the DeepDOM metatranscriptomes are denoted with a purple triangle. Black circles denote nodes with >80% bootstrap support (see Materials and Methods for details).

The divergent nature of the HMT quinoproteins makes substrate prediction for these enzymes extremely speculative. The most well-characterized of the PQQ-dependent enzymes are the membrane-bound glucose dehydrogenases (mGDH) and soluble methanol dehydrogenases (MDH). Both mGDH and alcohol dehydrogenases can act on a range of hexose and pentose sugars (64), with substrate flexibility potential determined by the opening of the active site in the β-propeller folds (65). We note that all HMT quinoproteins lack the disulfide cysteine-cysteine motif common to all bona fide MDH (62, 66), but that 4 variants do retain a Asp-Tyr-Asp (DYD) motif common to the lanthanide-dependent methanol dehydrogenases absent from other MDH forms (61), while another 5 retain a DXD motif without the conserved tyrosine (Fig. 5). Of the 21 putative PQQ-dehydrogenase families, fifteen contain signal peptides and nineteen contain predicted transmembrane domains, suggesting membrane localization (Fig. 5). It is remarkable that 21 full-length PQQ-dependent dehydrogenases would require ∼37 kbp to encode (assuming 1,800-bp genes that appears typical in HMT), providing a conservative estimate that ∼3% of the total genomic repertoire of the HMT is devoted to these enzymes alone. The large number of PQQ-dependent dehydrogenases together with the potential broad substrate specificity of these enzymes suggests that they target a diverse range of compounds and are an important component of HMT metabolism.

The PQQ dehydrogenase families present in the HMT genomes are all divergent from references available in the NCBI RefSeq database; of all 21 of these enzymes in our consolidated HMT set, the best match was query protein ASW8bin1_85_3 to a PQQ dehydrogenase in *Pseudomonas* sp. strain NBRC 111131 (accession no. WP_156358113.1), but even then the alignment had only 33.8% identity (Data Set S3). We also compared the HMT PQQ enzymes to the NCBI NR database, which recovered hits with higher percent identity in several uncultivated *Thaumarchaeota*, *Planctomyces*, *Euryarchaeota*, and *Chloroflexi* members (29 to 100% identity among the top 5 hits of each HMT enzyme; Data Set S3). Many of the reference proteins in the NCBI NR database are classified as hypothetical proteins, underscoring the divergent nature of these proteins compared to characterized enzymes in NCBI RefSeq (Data Set S3). We performed a phylogenetic analysis of the HMT PQQ-dependent dehydrogenases together with their best BLASTP hits in the NCBI NR database, which demonstrates that while many HMT enzymes cluster with those of other thaumarchaea, some cluster with proteins from *Chloroflexi*, *Planctomyces*, and *Euaryarchaeota*, suggesting that horizontal gene transfer has shaped the distribution of these enzymes to some extent (Fig. 5). Some of the HMT enzymes clustered together with proteins on fosmids sequenced from marine *Thaumarchaeota* in deep waters of the Ionian Sea (67) (KM3_90_H07 and AD1000-70-G10 in Fig. 5), indicating that these enzymes are present in other marine thaumarchaea.

The presence of a large-subunit RuBisCO homolog (*rbcL*) in the HMT genomes is a notable feature of this group. We identified this gene in 4 of 5 HMT MAGs (missing only in HMT_AABW); it is also present in the ASW8 MAG, as previously reported (24). A phylogenetic analysis of the HMT RuBisCO homologs placed it with other form III-a members of this protein family, albeit with long branches that demonstrate it is divergent from any previously identified homolog (Fig. S5a). To assess the likelihood that the HMT RuBisCO homologs are functional, we searched for 19 residues shown to be critical for the substrate binding and activity of this enzyme (68, 69) and successfully identified conservation of 17 of these residues (Fig. S5b). The two positions in the alignment where conserved residues were not conserved were 223 (aspartate instead of glycine) and 226 (tyrosine instead of phenylalanine or another hydrophobic residue), but a recent motif analysis of the RuBisCO protein family has shown that two positions tend to be among the most variable of the 19 conserved residues (70), indicating that activity is potentially retained despite these differences. Several recent studies have identified RuBisCO in disparate archaeal lineages; one study of Yellowstone hot spring metagenomes provided the first report of RuBisCO-encoding *Thaumarchaeota* (Beowolf and Dragon archaea) (17), where the authors postulated that this enzyme functions as part of an AMP-scavenging pathway. Several subsequent studies identified diverse RuBisCO in a large number of DPANN archaea (9, 70), and these studies further postulated that many of these enzymes participate in nucleotide scavenging, with one even demonstrating the activity of a form II/III version (71). However, AMPase, a key enzyme of the AMP salvage pathway responsible for generating R15P, could not be identified in the HMT genomes. This was also reported for the ASW8 MAG (24), where those authors suggested that R15P is generated from phosphoribosyl pyrophosphate by a Nudix hydrolase. Homologs for all genes in this proposed pathway cyclization are present in the HMT genomes (Fig. 4 and Data Set S4).

10.1128/mSystems.00415-20.5 FIG S5 (A) Maximum-likelihood phylogeny of RuBisCo large subunits. Reference sequences and RuBisCO form classifications were obtained from Jaffe and Banfield (70) (see Materials and Methods for details). (B) Sample alignment including different forms of RuBisCo, with arrows indicating 19 conserved residues shown to be important for enzymatic activity (see the main text). The two unfilled arrows indicate residues that differ between HMT RuBisCo and other forms. Download FIG S5, PDF file, 0.4 MB. Copyright © 2020 Aylward and Santoro. 2020 Aylward and Santoro This content is distributed under the terms of the Creative Commons Attribution 4.0 International license.

**FIG 6:**
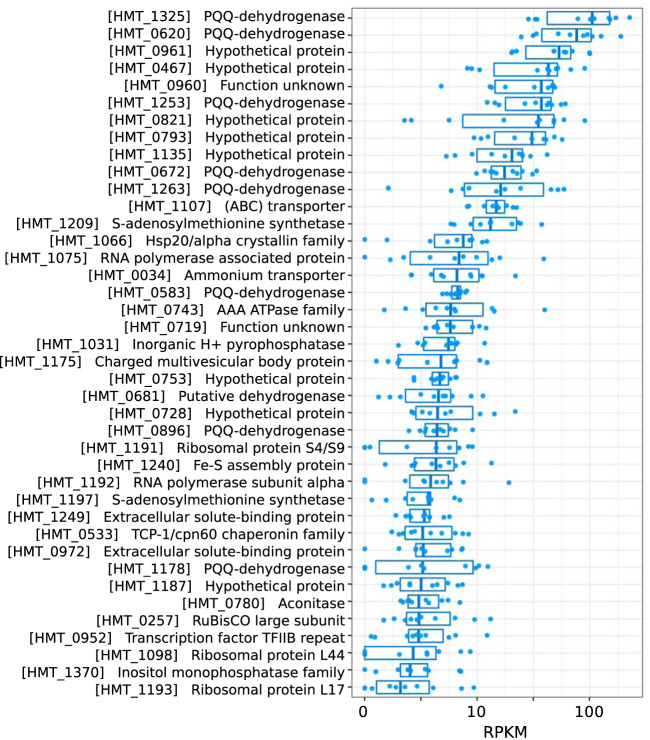
Top 40 HMT genes with highest median RPKM in 10 DeepDOM metatranscriptomes. Protein names refer to a nonredundant set of HMT proteins collectively present in the HMT MAGs. Full annotations for these proteins can be found in Data Set S3.

To provide additional insight into the metabolic priorities of the HMT lineage, we analyzed the *in situ* gene expression patterns in 10 metatranscriptomes collected at depths of 750 to 5,000 m in the South Atlantic during the DeepDOM cruise (Fig. 6 and Data Set S1; details are in Materials and Methods). The PQQ-dependent dehydrogenases were among the most highly expressed HMT genes across all samples, with eight among the top 40 most highly expressed HMT genes (denoted with purple triangles in Fig. 5), further highlighting the important role of these enzymes in the physiology of HMT. An ammonium transporter, ribosomal proteins, chaperones, and several hypothetical proteins were also among the most highly expressed (Fig. 6 and Data Set S1).

### Conclusions.

In this study, we characterized a group of putatively heterotrophic marine thaumarchaea (HMT) that is widespread in the global ocean. By analyzing MAGs from this group assembled from Atlantic and Pacific metagenomes, we show that they have small genomes and a predicted chemoorganoheterotrophic metabolism. Several unique features suggest adaptations to energy scarcity. The presence of numerous encoded PQQ-dependent dehydrogenases suggests the importance of oxidizing diverse carbon compounds and introducing reducing equivalents directly into the electron transport chain, which may be a critical component of their physiology in deep waters where energy is scarce. These PQQ dehydrogenases are among the most highly expressed genes in HMT and comprise up to ∼3% of the total base pairs in their genomes, underscoring their likely importance. Further, HMT encode a highly expressed form III-a RuBisCO that potentially functions as part of a CO_2_ incorporation pathway and may supplement organic carbon uptake for biosynthesis. Finally, a 2M-type complex I may allow HMT to pump an additional proton per electron and increase their overall energetic efficiency.

Ribosomal rRNA gene surveys have previously identified this group (28, 30), and closely related sequences are observed in diverse ocean provinces around the globe (25, 26, 29, 32–34). One study sequenced three fosmid sequences from the HMT group and noted that one encoded a PQQ-dependent dehydrogenase (30); however, these fosmids only had 41 to 73% amino acid identity and 85 to 95% 16S rRNA gene identity to our HMT genomes; therefore, they likely represent a distinct group within this lineage. This suggests that the MAGs we present here, which all have >95% average nucleic acid identity to each other, represent only a subset of the overall diversity of heterotrophic thaumarchaea in the ocean.

Although previous work on the marine *Thaumarchaeota* has been focused on chemolithoautotrophic ammonia-oxidizing lineages, our findings lead to the surprising conclusion that chemoorganoheterotrophic thaumarchaea are also widespread in the global ocean. In addition to broadening our understanding of archaeal diversity in the ocean, this finding could have multiple implications for biogeochemical cycling in the deep ocean. First, archaeal lipid distributions are used as paleoproxies of past ocean temperature. Current interpretations of the isotopic composition of archaeal lipids from marine sediments (e.g., see reference 72) and the water column (73) require up to 25% of archaeal carbon to be heterotrophic in origin or invoke variable isotopic fractionation (74). If heterotrophic HMT are, as our data suggest, as much as 6% of the planktonic AOA community, this could provide some of the heretofore “missing” heterotrophic signal in the archaeal lipid data. Second, the quinoprotein-facilitated oxidation of organic carbon compounds consumes O_2_ if electrons are passed through the entirety of the ETC but does not immediately yield CO_2_. If the products of these dehydrogenases are released and not further assimilated by the cell, this would lead to the consumption of deep ocean O_2_ that is not coupled to a corresponding consumption of dissolved organic carbon (DOC) and an anomalous respiratory quotient (75). Such cycling could provide a specific mechanism whereby DOC in the deep ocean decreases in lability with only minor changes in concentration (76). Further studies will be needed to examine the implications of the presence of these globally distributed thaumarchaea in the ocean.

## MATERIALS AND METHODS

### Metagenomes used for MAG construction.

We constructed MAGs from metagenomic data generated from the DeepDOM cruise in the South Atlantic in April 2013 (77, 78) and bioGEOTRACES samples collected during the Australian GEOTRACES southwestern Pacific section (GP13) in May to June 2011. Data from the bioGEOTRACES samples have been described previously (79), and only metagenomic data sets corresponding to depths of >4,000 m were examined here. DeepDOM metagenomes were generated using methods previously described (80), and metagenomes were processed through the Integrated Microbial Genomes/Microbiomes (IMG/M) workflow at the Joint Genome Institute (81). Metagenome samples correspond to the IMG/M accession numbers 3300026074, 3300026079, 3300026080, 3300026084, 3300026087, 3300026091, 3300026108, 3300026119, and 3300026253. DeepDOM samples were sequenced on an Illumina HiSeq 2500, and reads were subsequently assembled using SPAdes v. 3.11.1. To generate an HMT MAG from the South Pacific, we used reads from four deepwater metagenomes from the bioGEOTRACES samples (79) (SRA accession numbers SRR5788153, SRR5788420, SRR5788329, and SRR5788244).

### MAG construction.

We constructed MAGs from nine DeepDOM metagenomes using MetaBat v. 2.0 (82). We binned contigs in each metagenome using the following four different parameter sets: (i) -m 5000 -s 400000, (ii) -m 5000 -s 400000 -minS 75, (iii) -m 5000 -s 400000 -maxEdges 150 -minS 75, and (iv) -m 5000 -s 400000 -maxEdges 100 -minS 75 -maxP 90. Using this approach, we generated 1,166 total bins (many of which were redundant bins generated with different binning parameters), which we then evaluated using CheckM v 1.0.18 (83) (lineage_wf feature, default parameters). We then generated a set of dereplicated high-quality MAGs from each metagenome using dRep v. 2.3.2 (84) (dereplicate feature, CheckM results used as input, default parameters including 75% completeness and 5% contamination cutoffs). We did this for all nine DeepDOM metagenomes and recovered 66 total high-quality MAGs, of which 5 were HMT. Among the 5 HMT MAGs recovered, two had estimated completeness of >90% (5009_S15_AABW_high.53 and 2500_S23_NADW_med.34), and to generate a single high-quality reference MAG, we scaffolded these two MAGs together using Minimus2 in the AMOS package (85) (default parameters), which is similar to an approach used recently in a large-scale genomes-from-metagenomes workflow (86). Using this approach, we generated the HMT_ATL MAG, estimated to be 98% complete with 1.97% contamination by CheckM. For subsequent analysis, we analyzed the HMT_ATL MAG together with the HMT_AABW, HMT_AAIW, and HMT_NADW MAGs, which were binned directly from MetaBat2.

Having assembled high-quality HMT MAGs from deepwater Atlantic metagenomes, we sought to identify this group in similar samples from the Pacific; therefore, we analyzed four deepwater metagenome sequences as part of the bioGEOTRACES cruises (79). For these metagenomes, we used the same MAG binning approach that we had used for the DeepDOM samples, but we were unable to recover any HMT MAGs with completeness of >75% this way. Therefore, we used a coassembly approach for these samples. For this, we mapped reads from the four bioGEOTRACES samples mentioned above against the HMT_ATL MAG, given this was the highest quality reference, using bowtie2 v. 2.3.4.3 (default parameters [87]), and subsequently pooled and reassembled all mapped reads using SPAdes v. 3.13.1 (default parameters) (88). Using this approach, we recovered a high-quality HMT MAG, referred to as HMT_PAC, that has estimated completeness of 96.13% and contamination of 0.97% according to CheckM (Table 1).

### MAG annotation.

We calculated average nucleic acid identity (ANI) between MAGs using ANIcalculator v. 1 (89) (default parameters). We classified the HMT genomes using the Genome Taxonomy Database Toolkit v. 1.1.0 (default parameters) (21). We predicted genes and proteins from the HMT MAGs using Prodigal v 2.6.3 (default parameters) (90). We annotated proteins by comparing them to the EggNOG 4.5 (91) and Pfam 31.0 (92) databases using the hmmsearch command in HMMER v. 3.2.1 (93) (parameters -E 1e-5 for EggNOG and -cut_nc for Pfam), and we retrieved KEGG annotations using the KEGG KAAS server (single-direction method) (94). We predicted rRNAs on all MAGs using barrnap v. 0.9 (default parameters) (https://github.com/tseemann/barrnap). We predicted signal peptides using the SignalP-5.0 server (95), transmembrane topology using Phobius on the server (http://phobius.sbc.su.se/) (96), and transporters using the TransAAP server in the TransportDB 2.0 utility (97). Annotations from this pipeline for all MAGs can be found in Data Set S3 in the supplemental material. An additional automated functional annotation was conducted on the ATL MAGs using the Magnifying Genomes (MaGe) annotation pipeline (98) on the MicroScope platform (99) as part of a manual curation of HMT pathways. Annotations discussed in the text are based on the manual curation of the two best-quality HMG MAGs (HMT_ATL and HMT_PAC) while cross-referencing a nonredundant set of HMT proteins present in all MAGs (see “Nonredundant HMT protein set construction,” below). Structure prediction for annotation of the PQQ-dependent hydrogenases was conducted using RaptorX (http://raptorx.uchicago.edu/) (100). This manually curated subset of genes and pathways can be found in Data Set S4.

### Genome completeness estimates.

We estimated the completeness of the genomes in our study using two different approaches. First, we used CheckM v 1.0.18 (lineage_wf feature with default parameters), which used a set of 145 marker genes (k__Archaea [UID2] marker set). For an alternative approach, we used a set of 162 highly conserved single-copy markers previously used to assess completeness in archaea (38), which we refer to as the Rinke marker set. Because this set of markers was previously used for all archaea, as opposed to only the *Thaumarchaeota*, we first evaluated the presence of these markers in a set of 23 complete *Thaumarchaeota* genomes that are currently available (these genomes are present in our high-quality genome set; full set and marker annotations are in Data Set S2). We identified the presence of these markers in these genomes by searching their predicted proteins against the associated HMMs using hmmsearch (-cut_nc cutoff) and compiling the results. We found a set of 113 of these markers that was present in ≥22 of the 23 reference genomes used, and we used this set for subsequent estimation of genome completeness in our high-quality genome set (see “Reference genome sets used,” below; full results are in Data Set S2). MAG completeness and contamination estimates were used to extrapolate complete genome sizes using methods previously described (101).

### Reference genome sets used.

We generated multilocus concatenated protein phylogenies of the HMT MAGs using two sets of representative genomes as references, which we refer to as the full set and the high-quality set. For the full set, we included genomes with completeness of >50% and contamination of <5%. To obtain relevant reference genomes for this set, we downloaded all *Thaumarchaeota* available on NCBI as of 1 December 2019. We added available “*Candidatus* Aigarchaeota” and “*Candidatus* Geothermarchaeota” genomes available on the IMG/M database (81) at the same time; the latter two groups were included because they are known close relatives of the *Thaumarchaeota* and, therefore, can provide useful phylogenetic context (18, 102). When compiling this genome set, we also considered 3 *Thaumarchaeota* genomes that are available in IMG but not NCBI: the Dragon (DS1) and Beowolf *Thaumarchaeota* identified in geothermal springs (17), and the *Thaumarchaeota* fn1 genome found in anoxic peat (20). Lastly, for use as outgroup taxa, we also included three genomes from the *Crenarchaeota*: Sulfolobus islandicus L.S.2.15, Pyrobaculum aerophilum strain IM2, and Thermosphaera aggregans DSM 11486.

We generated a second set of genomes for use in ancestral state reconstructions, which we refer to as the high-quality set. For this, we selected a subset of genomes from the full set described above. In general we only selected genomes with completeness of >90% and contamination of <2% (estimated using the lineage_wf function in CheckM v 1.0.18 with default parameters), with two exceptions: the Dragon *Thaumarchaeota* DS1 and *Thaumarchaeota* fn1 had completeness and contamination estimates slightly outside the cutoffs used for the high-quality genome set (89% completeness for the Dragon *Thaumarchaeota* and 2.7% contamination for *Thaumarchaeota* fn1), but these genomes were included in this set nonetheless because they represent basal-branching *Thaumarchaeota* that are valuable for ancestral state reconstructions. The genomes in the high-quality set were chosen to represent as broad a phylogenetic breadth of *Thaumarchaeota* as possible and without overrepresenting any particular phylogenetic group, and to this end we manually curated this set to remove several AOA genomes if close relatives were still represented. A full list of the genomes included in these two sets can be found in Data Set S2.

### Molecular phylogenetics.

For phylogenetic reconstruction of the high-quality and full-genome sets, we used a set of 30 marker genes that includes 27 ribosomal proteins and 3 RNA polymerase subunits. These proteins have previously been benchmarked for concatenated phylogenetic analysis (103, 104). We predicted marker proteins using the markerfinder.py script, which is available on GitHub (https://github.com/faylward/markerfinder). This script uses a set of previously described Hidden Markov Models to identify highly conserved marker genes in genomic data (103). We generated a concatenated alignment of these markers using the ETE3 toolkit v. 3.1.1 (105) (workflow standard_trimmed_fasttree), which aligned the subunits with Clustal Omega v. 1.2.1 (106), trimmed the alignment with trimAl v. 1.4 (-gt 0.1 option) (107), and generated a diagnostic tree using FasTree v. 2.1.8 (108). We then constructed a maximum likelihood tree using IQ-TREE v. 1.6.6 (109) and assessed confidence with ultrafast bootstrap support (110). The model LG+F+I+G4 was the best-fit model inferred using the ModelFinder utility in IQ-TREE (111).

To assess the consistency of our phylogenetic reconstruction, we generated alternative phylogenies of the high-quality genome set using different marker proteins and phylogenetic models. For the first, we used the full set of 40 broadly conserved proteins described previously (103), which includes the 30 markers that we initially used in addition to several others, including multiple tRNA synthetases. For the second, we used only the 27 ribosomal proteins of our original set. For both of these phylogenies, we used IQ-TREE v. 1.6.6 with the appropriate model chosen with the ModelFinder utility and confidence assessed using 1,000 ultrafast bootstraps. For the third alternative phylogeny, we sought to assess the potential impact of the phylogenetic model on our results; therefore, we reconstructed a phylogeny using the original 30 marker proteins that we described above but with the C60 profile mixture model, which has been shown to be useful for phylogenetic estimation of deep-branching groups (112). These trees are available online at https://data.lib.vt.edu/files/fj236226r.

For the 16S rRNA gene tree, we obtained representative marine thaumarchaea sequences from NCBI GenBank (113) and the Ribosomal Database Project (RDP) (114). Reference sequences with high similarity to HMT 16S rRNA gene sequences were initially identified using the Classifier in RDP (115). We aligned reference and HMT 16S rRNA gene sequences using MAFFT (116) with the -localpair option, trimmed the alignment with trimAl (-automated1 option), and generated the tree with IQ-TREE using the SYM+G4 model, broadly consistent with previous approaches (3).

For the RuBisCO phylogeny we used reference RbcL proteins from a recent large-scale survey of this enzyme in genomic and metagenomic databases (70). We aligned the RbcL proteins from the HMT MAGs together with the references using Clustal Omega v. 1.2.3 (106) (default parameters), trimmed the alignment with trimAL (107) (parameter -gt 0.05), constructed a tree using IQ-TREE with the appropriate model chosen with the ModelFinder utility, and assessed confidence using 1,000 ultrafast bootstraps.

For the ATPase phylogeny, we generated a concatenated alignment of subunits A and B of this complex. These subunits were chosen because they are among the longest proteins in the ATPase operon, they are present in both the A-type and V-type operons as previously described (58), and they are generally colocated and can be easily identified. We downloaded Hidden Markov Models for subunits A and B from the EggNOG 4.5 database (COG1155 and COG1156, respectively) on 12 February 2020 and searched for these proteins in our high-quality genome set using hmmsearch (parameter -E 1e-10). We generated a concatenated alignment of these subunits using the ETE3 toolkit v. 3.1.1 (105) (workflow standard_trimmed_fasttree), which aligned the subunits with Clustal Omega v. 1.2.1 (106), trimmed the alignment with trimAl v. 1.4 (107), and generated a diagnostic tree using FastTree v. 2.1.8 (108). A final tree was then constructed using IQ-TREE with the appropriate model chosen with the ModelFinder utility and assessed confidence using 1,000 ultrafast bootstraps.

For the PQQ phylogeny, we identified representative proteins by searching all 21 PQQ-dependent dehydrogenases in the HMT nonredundant protein set against both the RefSeq v. 99 database (117) and NCBI NR database (118) (downloaded 17 April 2020) using the BLASTP command in the NCBI BLAST+ suite (v. 2.10.0+) (119) (parameters -evalue 1e-5 -max_target_seqs 5 -max_hsps 1). For the phylogeny, we consolidated and dereplicated the top 5 hits of each query protein against the NR database and then aligned them with the HMT proteins using Clustal Omega (default parameters). To root the tree, we used the characterized PQQ-dehydrogenase from Comamonas testosteroni (120). We trimmed the alignment using trimAL (parameter -gt 0.1), generated an alignment using IQ-TREE with the appropriate model chosen with the ModelFinder utility, and assessed confidence using 1,000 ultrafast bootstraps.

### Orthologous groups.

We predicted proteins from each genome in the high-quality genome set using Prodigal v. 2.6.3 (90) and subsequently generated orthologous groups using Proteinortho v. 6.06 (121) (-*P* = BLASTP option used). We selected a representative protein from each OG at random and used these for subsequent annotations. We used the hmmsearch command in HMMER3 to compare these proteins to EggNOG 4.5 (E value cutoff of 1e−5) and Pfam v. 31 (-cut_nc cutoffs) and the KEGG KAAS server to retrieve KO accession numbers, as described above for the genome annotations.

### Comparison of HMT MAGs with fosmids.

We calculated the average amino acid identity (AAI) of the HMT MAGs with three fosmids that were previously sequenced from marine *Thaumarchaeota* to assess if the fosmids belonged to a closely related lineage. We did this by calculating pairwise best LAST hits of the proteins encoded in the MAGs and fosmids and averaging the percent identity of these hits (LAST v. 959; -BlastTab option used). Full results can be found in Data Set S3. Comparison of the 16S rRNA genes encoded in fosmids and HMT genomes was done using the BLASTN tool in NCBI BLAST+ v. 2.10.0 (119) (default parameters).

### Nonredundant HMT protein set construction.

To quantify the abundance of the HMT group in metagenomes and HMT genes in metatranscriptomes, as well as for annotation purposes, we generated a nonredundant set of HMT proteins from 7 MAGs (the 5 MAGs generated in this study in addition to ASW8 and UBA57). This was done because these MAGs are highly similar (ANI of >95%); therefore, they have many similar or identical encoded proteins. Therefore, the consolidated nonredundant set of proteins from all HMT MAGs can be considered a representation of the pangenome of these closely related groups. For clustering the proteins, we used CD-HIT v. 4.6 (default parameters), with a combined file of all HMT proteins used as the input. Protein annotations were derived from the individual genome annotations (see “MAG annotation,” above). For read mapping, the proteins were then masked with tantan v. 13 (with the -p parameter) to prevent possible mapping to low-complexity sequences. Masked sequences were then formatted in a LAST database using the lastdb command from LAST v. 1060 (with the -p parameter). Subsequent mapping was done using the LASTAL command with the parameters -f BlastTab -u 2 -m 10 -Q 1 -F 15, which uses a translated mapping approach (i.e., DNA to amino acid), as previously described (122, 123).

To provide a comparison of HMT and AOA relative abundances, we also generated a nonredundant set of AOA proteins for comparison. For this, we used the same methods as those for the HMT MAGs, with proteins predicted from 147 reference AOA genomes used instead. These genomes were selected from our full genome set and represent genomes available in NCBI with estimated completeness of >50% and contamination of <5% (a list of the genomes used can be found in Data Set S2). Mapping against these AOA genomes was used only to obtain a general trend of AOA abundance (as in Fig. S3); for direct comparisons of HMT and AOA, we implemented read mapping to HMT-specific RbcL and AOA-specific AmoA protein sequences, which are single-copy markers that can be used to accurately compare the relative abundance of these two groups.

### Read mapping from metagenomes.

To estimate the global abundance and biogeography of HMT, we mapped raw reads from several metagenomic data sets onto the nonredundant set of HMT proteins. In addition to the DeepDOM and bioGEOTRACES metagenomes that we used for MAG construction (see “Metagenomes used for MAG construction,” above), we also mapped reads from mesopelagic metagenomes from the Tara Oceans expedition and 89 metagenomes generated from the waters of Station ALOHA at depths ranging from 25 to 1,000 m (37) (results are shown in Fig. 1 and 2). For the Station ALOHA samples we also mapped reads against a nonredundant set of AOA proteins for comparison (see “Nonredundant protein set for metagenome and transcriptome mapping,” above). For this approach, we used LASTAL (36) (parameters -m 10, -Q 1, -F 15) and only retained hits with bit scores of >50 and identity of >90%. Results for the Tara, DeepDOM, and bioGEOTRACES mapping are provided in Fig. 1, while results for the ALOHA mapping are provided in Fig. 2. Raw data for all mapping analyses are provided in Data Set S1. To compare the relative abundance of HMT and AOA, we also mapped reads to both the HMT RbcL protein and a selection of 31 AmoA genes from representative AOA in NCBI RefSeq, with only best hits retained. For this comparison, we normalized these relative abundances by the length of the *amoA* or *rbcL* gene of each reference protein to arrive at final units of reads per kilobase per million (RPKM), which we used for final comparisons. All values can be found in Data Set S1.

### Transcriptome mapping.

We analyzed 10 metatranscriptomes collected during the same DeepDOM cruise in which the metagenomes were collected. These samples correspond to IMG/M accession numbers 3300011314, 3300011304, 3300011321, 3300011284, 3300011290, 3300011288, 3300011313, 3300011327, 3300011318, and 3300011316. Metatranscriptomes were processed using methods previously described (80). We mapped reads from all metatranscriptomes onto a consolidated nonredundant set of HMT proteins (see “MAG annotation,” above), using LAST with parameters -m 10 -u 2 -Q 1 -F 15, and the reference database was first masked with tantan (124), using previously described methods (122). We only considered hits with bit scores of >50 and percent identity of >90%, and we processed LAST mapping outputs using previously described methods (123) and normalized transcript counts using the RPKM method (125). Detailed information can be found in Data Set S1.

### Ancestral state reconstruction.

We performed ancestral state reconstructions using the 5 HMT MAGs and representative genomes present in our high-quality genome set (see “Reference genome sets used,” above). We estimated the presence of OGs in internal branches of the *Thaumarchaeota* tree using the “ace” function in the “ape” package in R (126), with OG membership treated as a discrete feature. We constructed a binary matrix of extant OG membership from the Proteinortho output and subsequently inferred ancestral OG membership at each internal node on an ultrametric tree, which we constructed using the “chronos” function. We rounded log likelihoods to the nearest integer to infer the probability of OG presence/absence at each internal node.

### Data availability.

The MAGs described in this study have been deposited in DDBJ/ENA/GenBank and are associated with BioProject PRJNA636088. The genomes of the 5 HMT MAGs are also available on the Aylward Lab FigShare account: https://figshare.com/articles/HMT_MAGs/12252731. Nucleic acid sequences for the MAGs, protein predictions, alignments for phylogenies, and other data products are available on the VTechData archival platform: https://data.lib.vt.edu/files/5712m673w.
