# Supplementary figures and images for: Heterotrophic Thaumarchaea with Small Genomes Are Widespread in the Dark Ocean
Source: mSystems. 2020 Jun 16;5(3):e00415-20. doi: 10.1128/mSystems.00415-20 (PMC7300363; doi:10.1128/mSystems.00415-20)

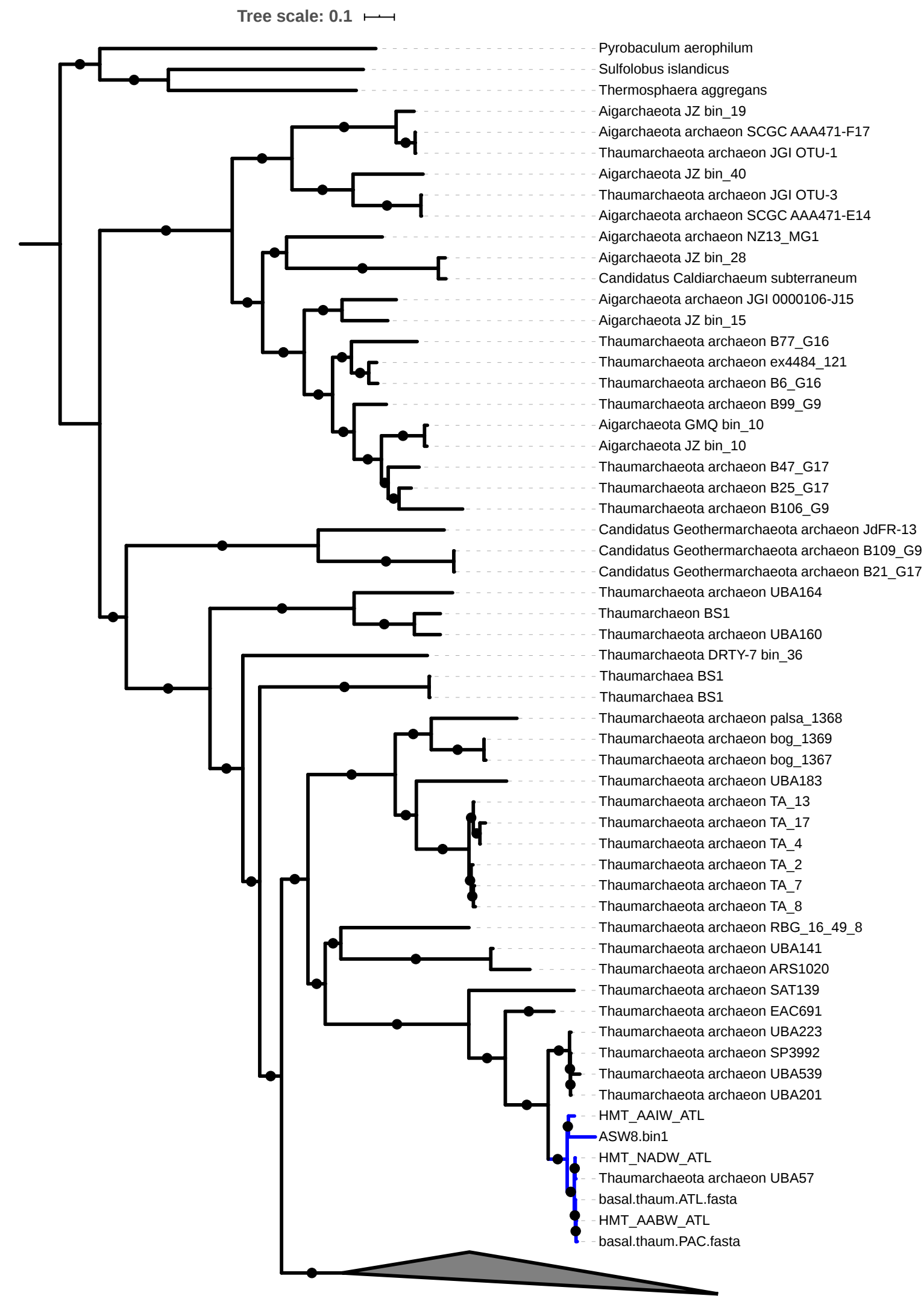

Supplement: FIG S1 [file mSystems.00415-20-sf001.pdf]

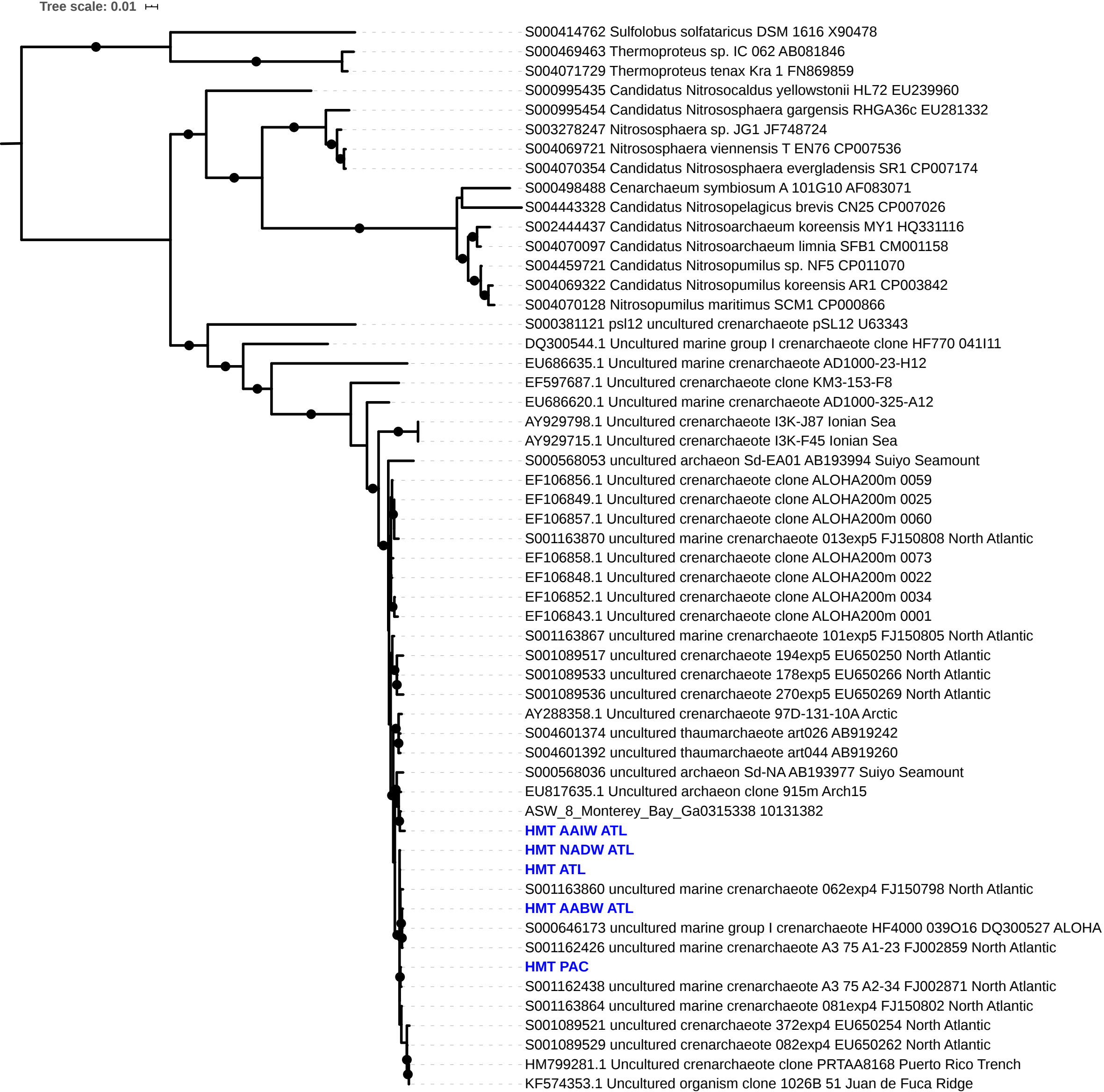

Supplement: FIG S2 [file mSystems.00415-20-sf002.pdf]

**HMT *rbcL* profile**

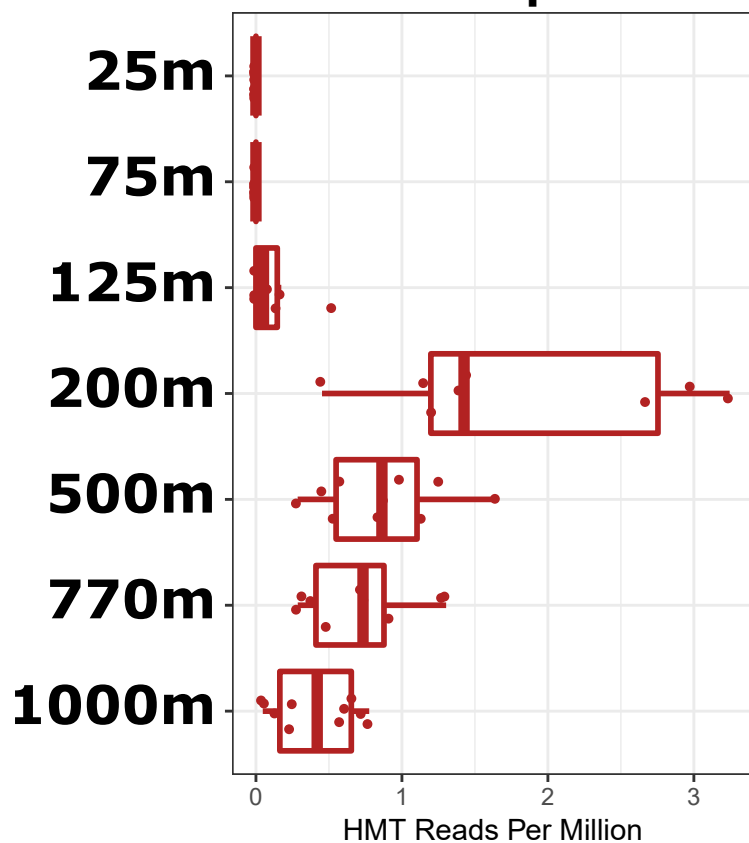

**AOA *amoA* profile**

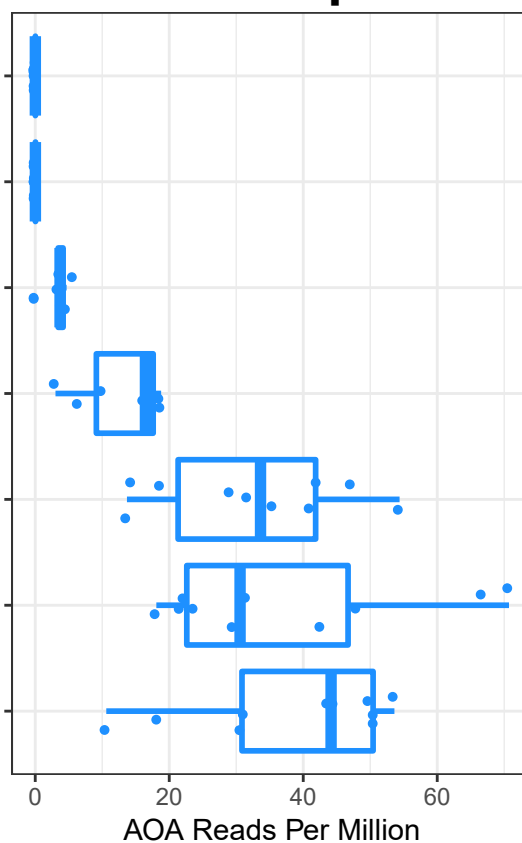

**HMT whole genome profile**

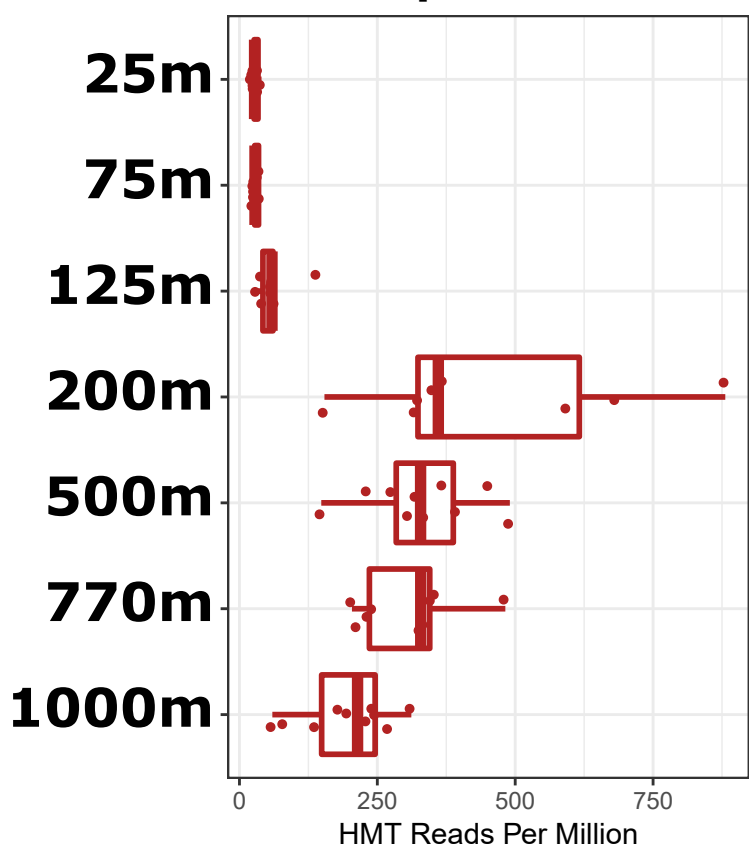

**AOA whole genome profile**

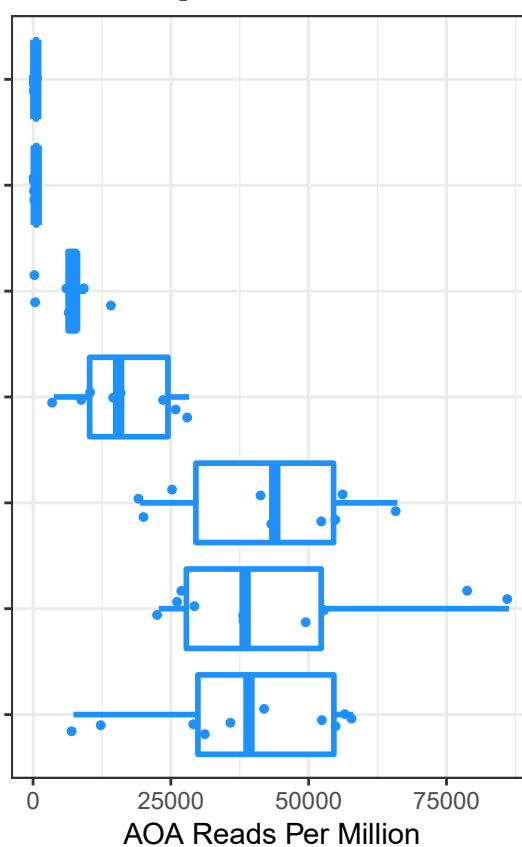

Supplement: FIG S3 [file mSystems.00415-20-sf003.pdf]

**A.**

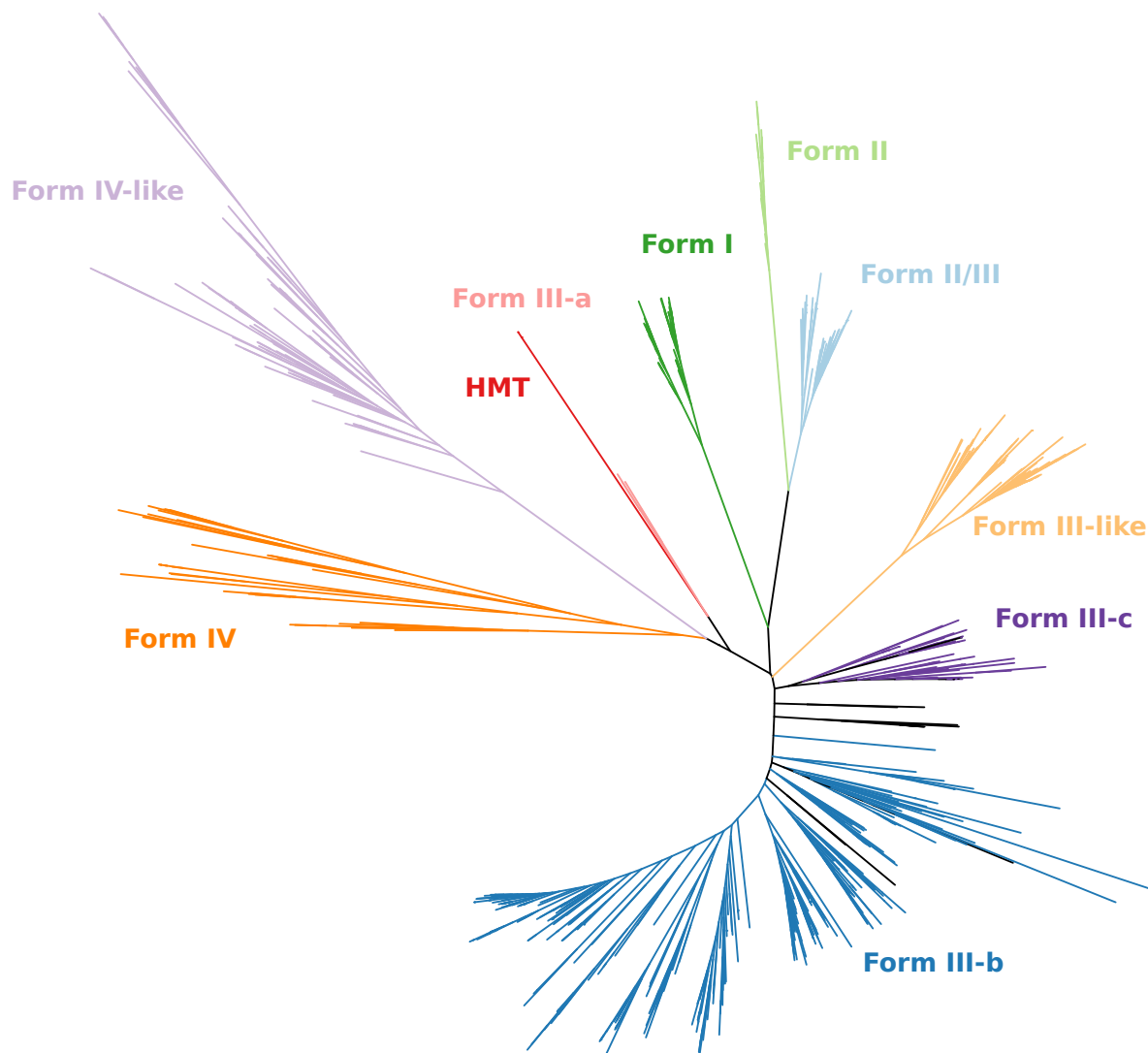

**B.**

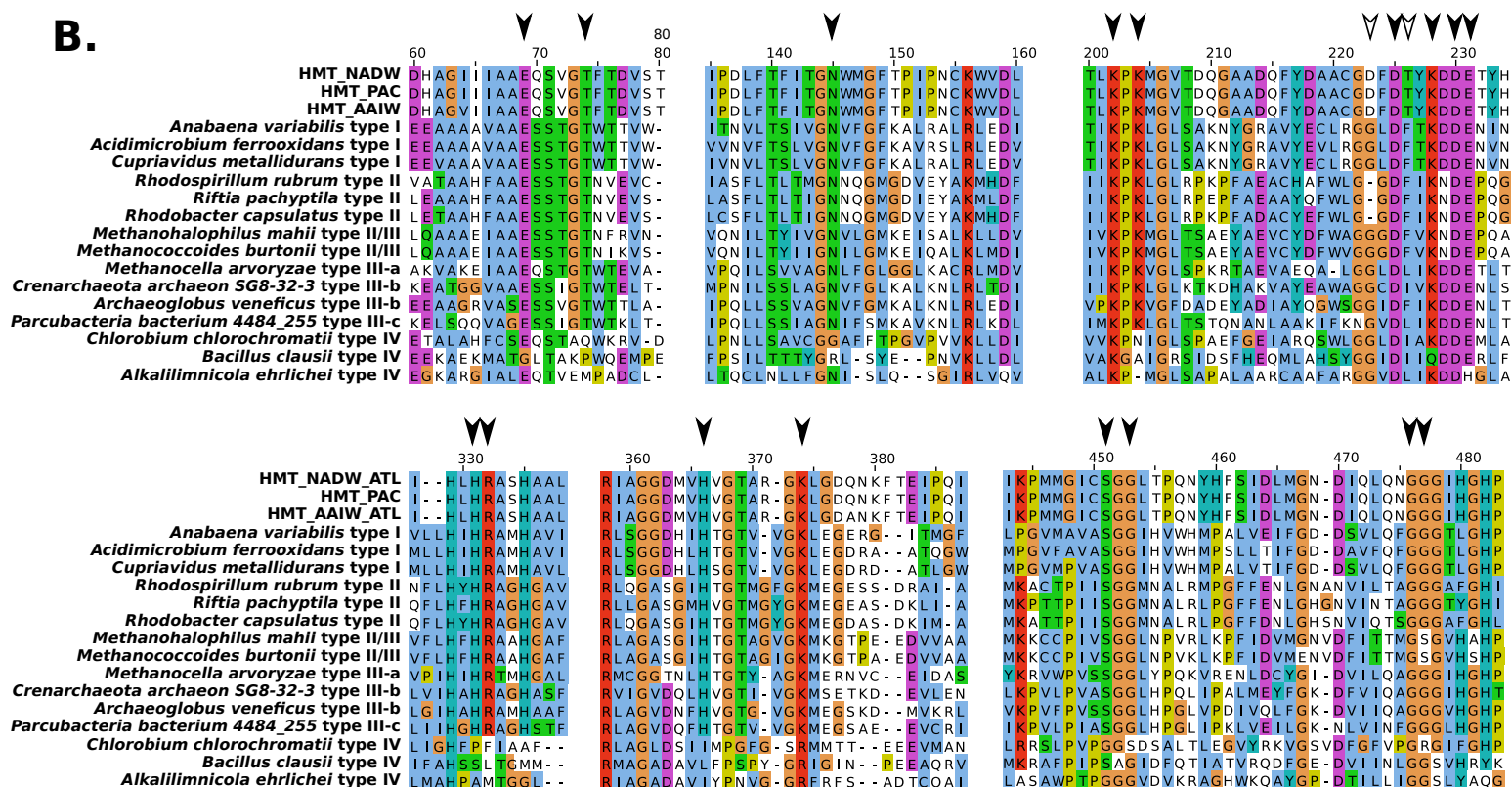

Supplement: FIG S5 [file mSystems.00415-20-sf005.pdf]
