# Supplementary material for: Heterotrophic Thaumarchaea with Small Genomes Are Widespread in the Dark Ocean
Source: mSystems. 2020 Jun 16;5(3):e00415-20. doi: 10.1128/mSystems.00415-20 (PMC7300363; doi:10.1128/mSystems.00415-20)
Supplement: FIG S4 [file mSystems.00415-20-sf004.pdf]

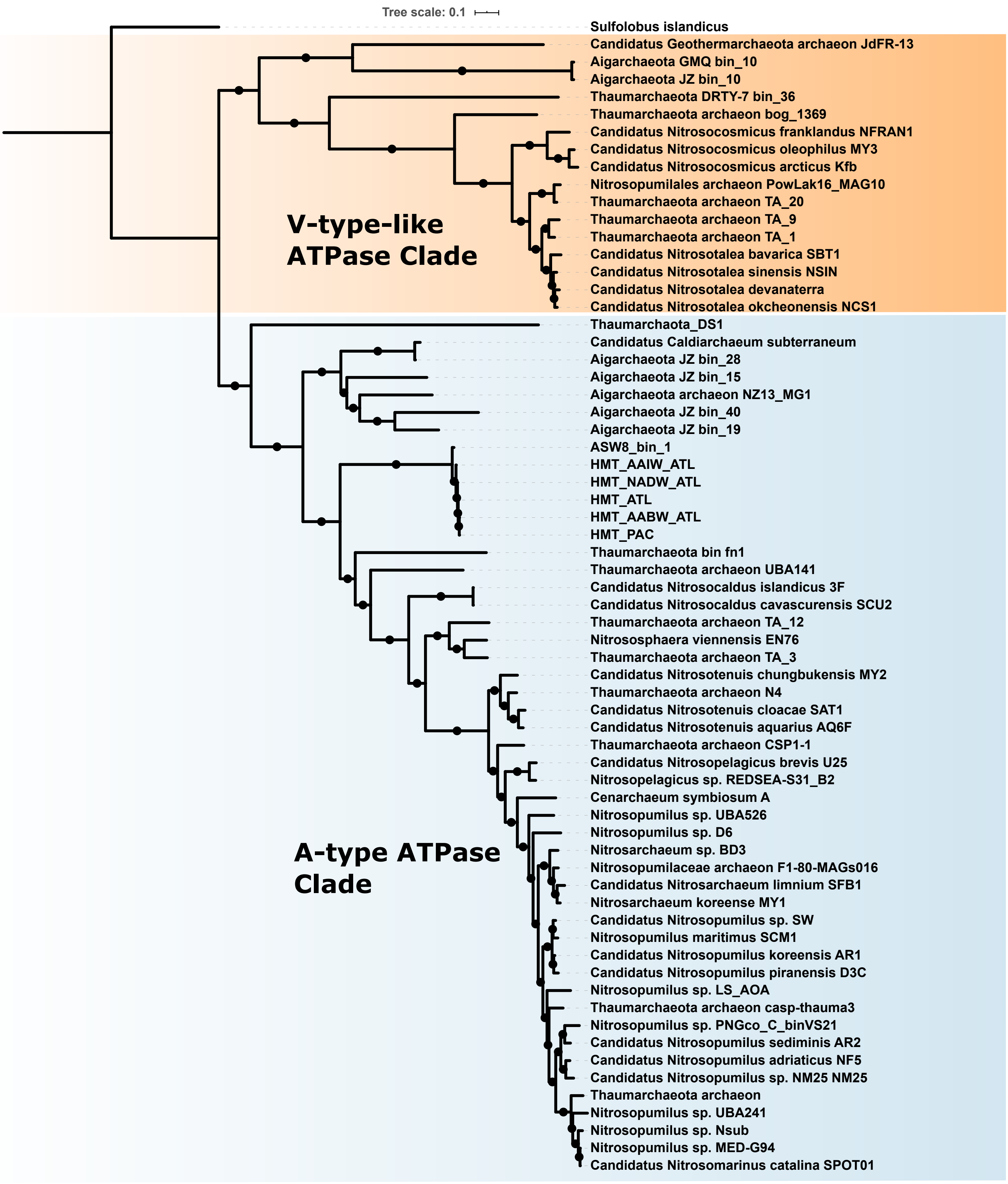

Tree scale: 0.1
V-type-like ATPase Clade
A-type ATPase Clade
Sulfolobus islandicus
Candidatus Geothermarchaeota archaeon JdFR-13
Aigarchaeota GMQ bin_10
Aigarchaeota JZ bin_10
Thaumarchaeota DRTY-7 bin_36
Thaumarchaeota archaeon bog_1369
Candidatus Nitrosocosmicus franklandus NFRAN1
Candidatus Nitrosocosmicus oleophilus MY3
Candidatus Nitrosocosmicus arcticus Kfb
Nitrosopumilales archaeon PowLak16_MAG10
Thaumarchaeota archaeon TA_20
Thaumarchaeota archaeon TA_9
Thaumarchaeota archaeon TA_1
Candidatus Nitrosotalea bavarica SBT1
Candidatus Nitrosotalea sinensis NSIN
Candidatus Nitrosotalea devanaterra
Candidatus Nitrosotalea okcheonensis NCS1
Thaumarchaota_DS1
Candidatus Caldiarchaeum subterraneum
Aigarchaeota JZ bin_28
Aigarchaeota JZ bin_15
Aigarchaeota archaeon NZ13_MG1
Aigarchaeota JZ bin_40
Aigarchaeota JZ bin_19
ASW8_bin_1
HMT_AAIW_ATL
HMT_NADW_ATL
HMT_ATL
HMT_AABW_ATL
HMT_PAC
Thaumarchaeota bin fn1
Thaumarchaeota archaeon UBA141
Candidatus Nitrosocaldus islandicus 3F
Candidatus Nitrosocaldus cavascurensis SCU2
Thaumarchaeota archaeon TA_12
Nitrososphaera viennensis EN76
Thaumarchaeota archaeon TA_3
Candidatus Nitrosotenuis chungbukensis MY2
Thaumarchaeota archaeon N4
Candidatus Nitrosotenuis cloacae SAT1
Candidatus Nitrosotenuis aquarius AQ6F
Thaumarchaeota archaeon CSP1-1
Candidatus Nitrosopelagicus brevis U25
Nitrosopelagicus sp. REDSEA-S31_B2
Cenarchaeum symbiosum A
Nitrosopumilus sp. UBA526
Nitrosopumilus sp. D6
Nitrosarchaeum sp. BD3
Nitrosopumilaceae archaeon F1-80-MAGs016
Candidatus Nitrosarchaeum limnium SFB1
Nitrosarchaeum koreense MY1
Candidatus Nitrosopumilus sp. SW
Nitrosopumilus maritimus SCM1
Candidatus Nitrosopumilus koreensis AR1
Candidatus Nitrosopumilus piranensis D3C
Nitrosopumilus sp. LS_AOA
Thaumarchaeota archaeon casp-thauma3
Nitrosopumilus sp. PNGco_C_binVS21
Candidatus Nitrosopumilus sediminis AR2
Candidatus Nitrosopumilus adriaticus NF5
Candidatus Nitrosopumilus sp. NM25 NM25
Thaumarchaeota archaeon
Nitrosopumilus sp. UBA241
Nitrosopumilus sp. Nsub
Nitrosopumilus sp. MED-G94
Candidatus Nitrosomarinus catalina SPOT01
